# Impact of fiber-optic laryngoscopy on the weaning process from mechanical ventilation in high-risk patients for postextubation stridor

**DOI:** 10.1097/MD.0000000000005971

**Published:** 2017-02-03

**Authors:** Malcolm Lemyze, Emmanuelle Durville, Mehdi Meddour, Marie Jonard, Johanna Temime, Stéphanie Barailler, Didier Thevenin, Jihad Mallat

**Affiliations:** a Department of Respiratory and Critical Care Medicine, Schaffner Hospital, Lens; b Intensive Care Unit, Arras Hospital, Arras, France.

**Keywords:** intensive care, laryngeal injury, mechanical ventilation, stridor

## Abstract

The objective of this study was to assess the impact of fiber-optic laryngoscopy (FOL) on the weaning process from mechanical ventilation in critically ill patients with a positive cuff leak test (CLT) as compared with the current recommended strategy based on corticosteroids.

In this prospective observational pilot study conducted over a 1-year period in a 15-bed ICU, CLT was systematically performed before extubation in all intubated patients having passed a spontaneous breathing trial (SBT). After the endotracheal tube cuff was deflated, cuff leak volume (CLVol) was assessed during assisted controlled ventilation. When CLT was positive (CLVol < 110 mL), patients either were evaluated using FOL by our half-time FOL-practitioner when present, or received corticosteroids.

Among the 233 patients included, 34 (14.6%) had a positive CLT that hampered extubation. Seventeen were treated by corticosteroids and 17 were evaluated by FOL. In the corticosteroids group, the CLVol was still <110 mL at 24 hours in 9 patients (53%). Corticosteroids strategy merely prolonged the total duration of mechanical ventilation (7 [4–11] vs 4 [2–6] days, *P* = 0.01) by increasing the time between successful SBT and the moment when extubation was effectively achieved (30 [24–60] vs 1.5 [1–2] hours, *P* < 0.001). This resulted in 2 self-extubations (12%) and 9 FOL-guided extubations (53%) in the corticosteroids group. Massive swelling of the arytenoids was the most common feature shown by FOL. The patients evaluated by FOL who exhibited the thin anterior V-shaped opening of the vocal cords—the V sign—(n = 26, 100%) were immediately extubated without any stridor or respiratory failure afterward.

In this pilot study, a FOL-based extubation strategy was feasible and reliable, and significantly reduced the duration of mechanical ventilation in patients with a positive CLT. We describe the “V sign” of FOL that safely allows a successful prompt extubation in patients considered at high risk for postextubation stridor.

## 1 Introduction

Laryngeal injuries following prolonged periods of invasive mechanical ventilation have been reported as a frequent cause of postextubation weaning failure in critically ill patients.^[1–4]^ Many efforts have been made to predict the risk of obstructive laryngeal edema that can lead to postextubation stridor and acute respiratory failure with a potential for the dreadful “no ventilate no airway” scenario. The cuff leak test (CLT) is commonly considered the best simple clinical test to predict the risk of stridor following extubation.^[5]^ It consists in measuring the leak through the intubated patient's airways once the cuff of the endotracheal tube is deflated. A negative CLT means that the leak volume is enough to ensure a low risk of postextubation stridor. On the opposite, a positive CLT refers to a reduced leak volume and is associated with a higher risk of postextubation stridor. CLT has been shown to have a high negative predictive value but a low specificity and overall a very low positive predictive value for predicting postextubation respiratory failure that requires urgent reintubation.^[5,6]^ One potentially harmful misconception is the intuitive link that is commonly made between a positive CLT and obstructive laryngeal edema that can cause such a dramatic scenario. Based on meta-analysis,^[7–9]^ experts have recommended the use of repeated doses of corticosteroids in patients with a positive CLT. Extubation is then reconsidered after 24 to 48 hours of high doses of corticosteroids. This strategy may merely delay the weaning process from mechanical ventilation and thus may prolong the hospital stay in very frail critically ill patients. Actually, no study has ever assessed the anatomical significance of a positive CLT. The latter does not necessarily mean that airflow is severely limited through the upper airways because of a massive swelling of the vocal cords. Other structures may be involved including the trachea and the arytenoids, or the patient's airflow may be limited simply by the combination of a thin glottis and a large endotracheal tube. We are convinced that the only way to be sure of the diagnosis is to perform a fiber-optic examination of the pharyngolaryngeal structures before extubation. This pilot study tests a new strategy using fiber-optic laryngoscopy (FOL) as compared with the usually recommended corticosteroids therapy.

## 2 Materials and methods

### 2.1 Patients

This prospective observational study was conducted at the Department of Critical Care Medicine of the Schaffner Hospital during a 1-year period, to evaluate all consecutive critically ill patients requiring invasive mechanical ventilation. The Schaffner Hospital ethics committee approved the study (approval number 150602) and signed informed consent was obtained from all the patients or next of kin.

Patients who failed the spontaneous breathing trial (SBT) or who had any of the following before SBT and CLT—tracheostomy, unplanned extubation including self-extubation and accidental extubation, or current pregnancy—were excluded from the study.

### 2.2 Protocol

All patients were intubated with a cuffed endotracheal tube (Mallinckrodt, Athlone, Ireland) with an inner diameter varying from 7.0 to 8.5 mm. All of them were without sedation, awake, and comfortably sit in the cardiac chair position. They had passed a-30 minute SBT and were ready to be extubated according to the attending physician. Our protocol-driven strategy is based on current recommendations^[10]^ as opposed to a planned extubation strategy, which has been shown to uselessly prolong the weaning process from mechanical ventilation.^[11]^ Patients who were ventilated in the pressure-cycled mode were transiently switched to assisted volume control mode ensuring that the volume delivered by the ventilator was constant from 1 respiratory cycle to another. In that case, tidal volume and inspiratory flow were set the closest as possible to the one generated by the patient in bilevel positive pressure ventilation. This strategy aims at preventing patient-ventilator dysynchrony and at maximizing patient's comfort during the CLT maneuver. Oral and endotracheal suctioning were performed before the cuff was deflated. Cuff leak volume was determined using the Miller formula as the difference between inspiratory volume before the cuff was deflated and the mean expiratory volume after the cuff's deflation. Mean expiratory volume was calculated as the average of the 3 lowest expiratory volumes of the patient during the first 6 respiratory cycles immediately following the cuff's deflation. CLT was considered positive when the cuff leak volume was less than 110 mL.^[5]^ When the CLT was positive, a FOL was performed by the same investigator if available as soon as possible. The latter was a half-time consultant in respiratory and critical care medicine who had practiced fiber-optic examination in critically ill patients for more than 10 years. When the FOL practitioner was not in the ICU, the patient received corticosteroids as recommended^[7]^ —methyl prednisolone 20 mg every 4 hours—and the CLT was performed again once a day until the cuff leak volume became greater than 110 mL. After 24 hours of corticosteroids therapy, if the CLT was still positive, the attending physician could decide to resort to a fiber-optic examination of the larynx to shorten the weaning process from mechanical ventilation.

Postextubation stridor (PES) was classically defined as an audible high-pitched inspiratory wheeze complicated by a respiratory distress needing medical intervention.^[7,12]^ Extubation failure refers to the need to reintubate the patient in the first 48 hours following extubation or to resort to palliative care in the case of do-not-reintubate orders.

### 2.3 Fiber-optic evaluation of the larynx

**Figure 1:**
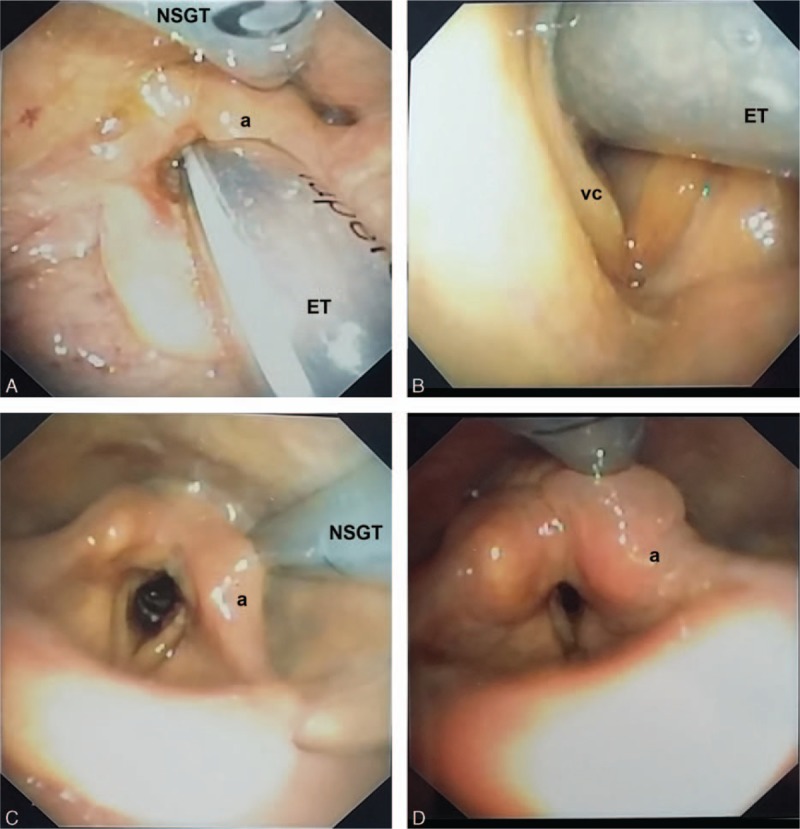
A, Massive swelling of the arytenoids (a) compressed between the nasogastric tube (NSGT) backward and the endotracheal tube (ET) forward. B, A good fiber-optic laryngoscopy practitioner will visualize the V sign—the V-shaped opening of the anterior part of the vocal cords (vc)—allowing extubation. C, Picture of the swollen posterior larynx at inspiration after successful extubation. D, Same case at expiration.

Under topical anesthesia with 4% lidocaine, a flexible fiber-optic bronchoscope (Olympus P160) was passed nasally to evaluate the posterior pharyngeal area, the arytenoid cartilage, the vocal cords, and their relationship with the endotracheal tube and the nasogastric tube. The presence of edema (localized or diffuse), ulcerations (superficial covered with mucosa or deep), and granulation was recorded. Each time the thin anterior V-shaped opening of the vocal cords—the “V sign” (Fig. 1B)—could be visualized just forward the endotracheal tube, the patient was immediately extubated. After extubation, the previous examination was completed by notification of ulcerations on the two-thirds posterior parts of the vocal cords (corresponding to the footprints of the endotracheal tube on the vocal cords), obstruction of the laryngeal lumen, abnormal vocal cords mobility, and granuloma, as previously described.^[3,4]^

### 2.4 Statistical analysis

Data are expressed as mean ± SD when they are normally distributed, or as median [25–75%, interquartile range (IQR)] when they are non-normally distributed. Proportions were used as descriptive statistics for categorical variables. The normality of data distribution was assessed using the Kolmogorov–Smirnov test. Comparisons of values between groups of patients were performed by the 2-tailed Student *t* test, or the Mann–Whitney *U* test, as appropriate. Analysis of the discrete data was performed by *χ*^2^ test or Fisher exact test when the numbers were small. Statistical analysis was performed using STATA 14.0 (StataCorp LP, College Station, TX). *P* <0.05 was considered statistically significant. All reported *P* values are 2-sided.

## 3 Results

In the current population of intubated critically ill patients (n = 233), 14.6% of the patients (n = 34) were considered at high risk of postextubation stridor according to a positive CLT. Briefly, patients with positive CLT (Table 1) were most likely female (n = 26, 76.5% vs n = 62, 31%; *P* < 0.001) with higher BMI (29 [26–34] kg·m^−1^ vs 26 [23–31] kg·m^−1^; *P* = 0.04) admitted for acute respiratory failure (n = 12, 35% vs n = 39, 20%; *P* = 0.05) as compared with those with negative CLT. They were more often treated by corticosteroids before extubation (n = 19, 61% vs n = 18, 9%; *P* < 0.001) and required longer time of mechanical ventilation (6 [3–9] days vs 3 [2–6] days; *P* < 0.001, respectively).

**Table 1:**
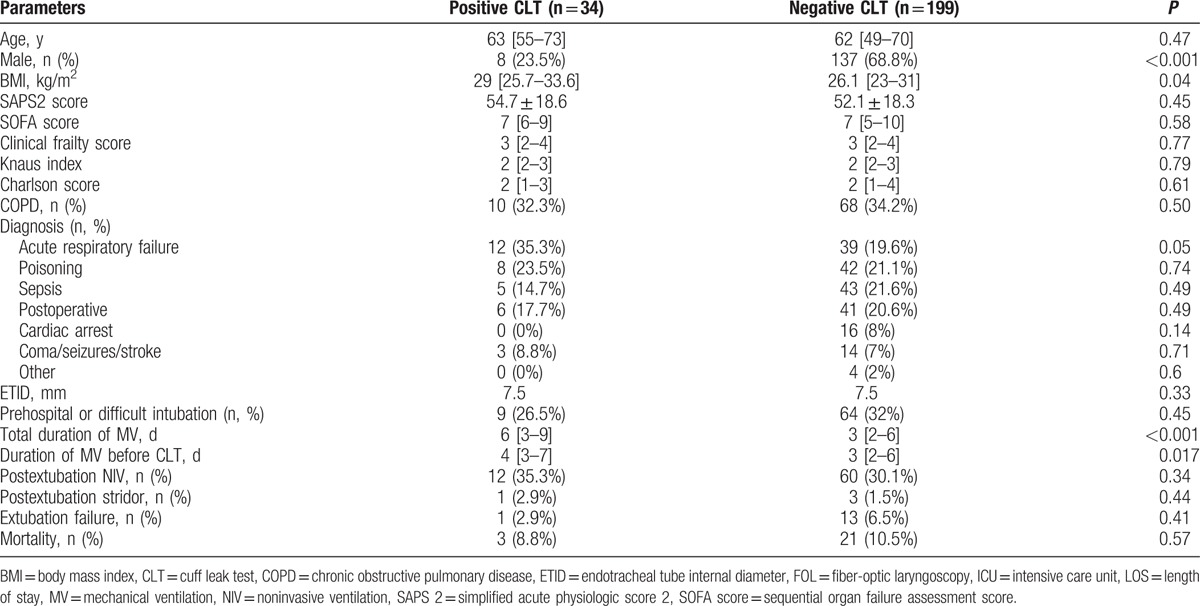
Main characteristics of the patients at high risk for postextubation stridor compared with those of the patients with negative cuff leak test.

Among the 34 patients with a positive CLT (14.6%) that hampered extubation, 17 patients were evaluated using FOL and 17 received corticosteroids. The 2 groups were similar (Table 2). In the corticosteroids group (n = 17), the cuff leak volume barely increased at 24 hours (22 [0–86.3] vs 97 [0–208] mL, *P* = 0.05) and was still <110 mL in 9 patients (53%). Corticosteroids strategy merely prolonged the total duration of mechanical ventilation (7 [4–11] vs 4 [2–6] days, *P* = 0.01) by increasing the time between successful SBT and the moment when extubation was effectively achieved (30 [24–60] vs 1.5 [1–2] hours, *P* < 0.001). This resulted in 2 self-extubations (12%) and 9 FOL-guided extubations (53%) in the corticosteroids group as requested by the attending physician after failure of the corticosteroids strategy to allow extubation after 24 hours. As shown in Table 3, in all the patients evaluated (n = 26), FOL revealed massive swelling of the posterior larynx mainly located on the posterior commissure and the arytenoids pinched between the nasogastric tube and the endotracheal tube (Fig. 1A). However, all the patients (n = 26, 100%) exhibited the V sign (Fig. 1B) allowing immediate extubation according to our protocol, and none of them developed any stridor or respiratory failure afterward.

**Table 2:**
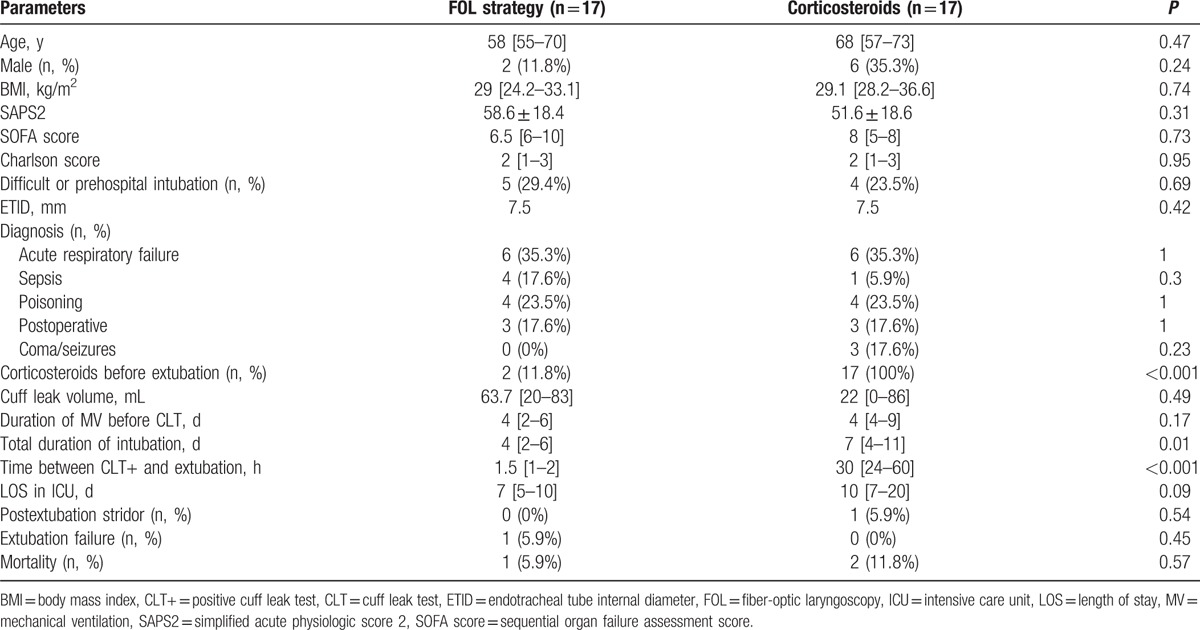
Main characteristics of the high-risk patients for post-extubation stridor according to the chosen therapeutic strategy, fiber-optic laryngoscopy versus corticosteroids.

**Table 3:**
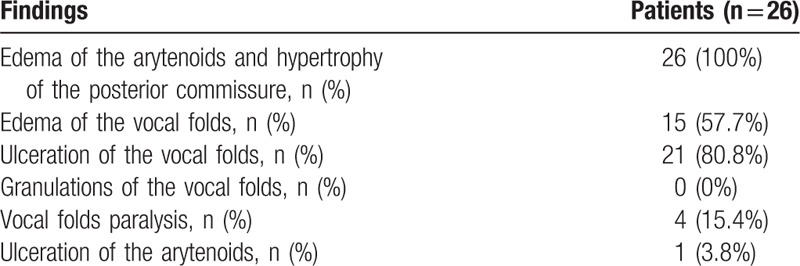
Main findings at fiber-optic laryngoscopy.

## 4 Discussion

The main results of the present study can be summarized as follows: corticosteroids strategy usually failed at increasing the cuff leak volume and delayed extubation; fiber-optic laryngoscopy revealed injuries of the posterior larynx with a major edema of the arytenoids rather than a swelling of the vocal cords; despite major edema of the posterior larynx at FOL, the “V sign,” when present, allowed safe early extubation with good outcome.

Corticosteroids have been proposed as a systematic therapeutic regimen to reduce the risk of postextubation laryngeal edema.^[6,7]^ In the large randomized controlled study of François et al,^[12]^ corticosteroids given 12 hours before a planned extubation significantly reduced the incidence of laryngeal edema as compared with a control group with a 22% incidence of the disease. The extremely high incidence of laryngeal edema in the control group of this study may be questionable. Actually, the diagnosis was truly confirmed by laryngoscopy in only 14 patients (4%). The problem is that, in many studies like the one of François et al, the definition of laryngeal edema comes down to the simple presence of postextubation stridor. From a physiological point of view, inspiratory dyspnea in a critically ill patient can result from end-expiratory gas trapping or abnormal postinspiratory abdominal muscle contraction—that both promote high auto-positive end expiratory pressure (auto-PEEP)—rather than the direct symptom of an occluded upper airway. Auto-PEEP acts as an elastic threshold load that must be overcome by the inspiratory muscles before the inspiratory effort can generate tidal volume.^[13]^ It can be assumed that PES may be abusively diagnosed in patients with COPD, morbid obesity, high intra-abdominal pressure, patient-ventilator asynchrony, or difficult weaning from mechanical ventilation, for whom the main cause of inspiratory dyspnea is auto-PEEP. On the other hand, planned extubation merely prolongs the weaning process from mechanical ventilation compared with a protocol-driven strategy and this is no longer acceptable today.^[12]^ Furthermore, repeated doses of corticosteroids place critically ill patients at high risk of developing delirium,^[14]^ dysglycemia,^[15]^ muscle weakness,^[16]^ immunosuppression, and superinfections.^[15]^ All of which are critical conditions associated with increased in-hospital mortality and higher morbidity during and after the ICU.^[14,16]^ Given the poor efficacy of high doses of corticosteroids at increasing the cuff leak volume in our population and the numerous adverse events induced by corticosteroids in the critically ill patient, it is very unlikely that this therapeutic strategy can safely prevent the risk of postextubation stridor without uselessly delaying extubation.

Major edema of the arytenoids and aryepiglottic folds was the most frequent feature revealed by fiber-optic examination of the upper airways in critically ill patients with a positive CLT. Several mechanisms may promote such a swelling of specific parts of the posterior larynx. Traumatic intubation especially when performed in an emergency setting is the most commonly recognized cause of laryngeal injuries in mechanically ventilated patients. Rapid sequence intubation—referring to the use of a neuromuscular blocking agent combined with a sedative—can facilitate the view of the larynx during direct laryngoscopy and thus reduce the risk of complications.^[3,17]^ Given their close position, endotracheal tube and nasogastric tube per se may damage the laryngeal structures (Fig. 1A). When pushed to extremes, this mechanism can lead to nasogastric tube syndrome. By exerting a prolonged extrinsic compression on the posterior cricoarytenoid muscle, the nasogastric tube can rarely cause ulceration and ischemia of this muscular structure resulting in a dramatic paralysis of the vocal folds in adduction.^[18]^ Finally, severe gastro-esophageal reflux (GER) is a classic provider of voluminous edema of the arytenoids and hypertrophy of the posterior commissure.^[19]^ Many conditions that promote GER—such as recumbent position, obesity, high intra-abdominal pressure, delayed gastric emptying, recent abdominal surgery, drug-induced nausea, for instance—are very common in the ICU and explain why critically ill patients are particularly at high risk of developing GER.^[20]^ The present study is not designed to demonstrate a cause–effect relationship between GER and positive CLT. However, the picture revealed by FOL in most of the patients with a positive CLT strongly supports this hypothesis.

Several limits have to be acknowledged. First, considering the nonrandomized design of the present study, selection bias cannot be ruled out. This prospective observational pilot study is a picture of what happens in the real life of an ICU. To reduce interobserver variability, a single practitioner performed all the FOL. The cuff leak volume was measured by the attending intensivist, and the choice to resort to corticosteroids or to FOL-guided strategy laid only on the presence or not of the practitioner skilled at FOL. Second, indirect laryngoscopy is often considered challenging in the intubated patient. Copious secretions often complicate the procedure. Most of the clinicians will fail to properly visualize the larynx of critically ill patients with standard flexible laryngoscopes. We always use a flexible bronchoscope with a higher suctioning capacity—thanks to a large suctioning channel—than the one of the devices used by our ENT colleagues. Finally, the results of this single-center study should be confirmed by a large multicenter trial but question the current recommended strategy based on corticosteroids only.

In this pilot study, a FOL-based extubation strategy was feasible and reliable, and significantly reduced the duration of mechanical ventilation in patients with a positive CLT. We describe the “V sign” of FOL that safely allows a successful prompt extubation in patients considered at high risk for postextubation stridor.
